# Effect of Breastfeeding on Childhood BMI and Obesity: The China Family Panel Studies

**DOI:** 10.1097/MD.0000000000000055

**Published:** 2014-08-22

**Authors:** Huiquan Jing, Hongwei Xu, Junmin Wan, Yang Yang, Hua Ding, Minyan Chen, Lizhuo Li, Ping Lv, Jingwei Hu, Jingyun Yang

**Affiliations:** Institute of Social Science Survey, Peking University, Beijing (HJ, HD, MC, PL, JH); Department of Social Science (HJ); Section of Discipline Construction and Development Planning (YY), Shenyang Medical College; Emergency Department (LL), Shengjing Hospital, China Medical University, Shenyang, Liaoning, China; Institute for Social Research (HX), University of Michigan, Ann Arbor, Michigan; Graduate School of Economics (JW), Fukuoka University, Fukuoka City, Fukuoka, Japan; and Rush Alzheimer’s Disease Center and Department of Neurological Sciences (JY), Rush University Medical Center, Chicago, Illinois.

## Abstract

The objective of this study is to investigate the effect of breastfeeding on childhood obesity in China.

We used data collected from the China Family Panel Studies, an ongoing, prospective, and nationwide longitudinal study to explore the extensive and dynamic social changes in China. A total of 7967 children were included in the analysis. Duration of breastfeeding was first treated as a continuous variable and subsequently dichotomized into ever versus never, ≥6 months versus <6 months, ≥8 months versus < 8 months, and ≥12 months versus <12 months. Multiple imputation was conducted and regressions with propensity score matching were performed. We also performed quantile regression to examine whether breastfeeding has an effect on childhood obesity among children with a specific quantile of body mass index (BMI).

Consistent with findings from recent studies, in both adjusted and adjusted regressions, we did not find any statistically significant effect of breastfeeding on reducing the risk of obesity (unadjusted odds ratio, OR = 1.02, 95% confidence interval, CI 0.99, 1.05, *P* = 0.12; adjusted OR 1.01, 95% CI 0.98, 1.05, *P* = 0.36) or excessive weight (unadjusted OR = 1.01, 95% CI 0.99, 1.03, *P* = 0.26; adjusted OR = 1.00, 95% CI 0.98, 1.02, *P* = 0.90). Results were similar using various dichotomization of duration of breastfeeding. Quantile regression revealed that longer duration of breastfeeding is associated with higher BMI among children with small to medium quantile of BMI.

Our findings echo recent research and caution against any population-wide strategy in attempting to reduce overweight and obesity through promotion of breastfeeding.

## INTRODUCTION

Childhood obesity is a major public health problem. In the United States, it is estimated that approximately 32% of children and adolescents aged 2–19 are overweight and about 17% are obese,^1^ more than double than in the 1970s. Children who have obesity after 6 years are 1.5 times more likely to develop adult obesity, irrespective of their parents’ obesity status.^2^ Obese youth are more likely to have cardiovascular risk factors, with an estimated 70% of obese youth having at least 1, and nearly 40% having at least 2, risk factors for cardiovascular disease.^3^ Obese children are at increased risk of a variety of other diseases, including insulin resistance, type 2 diabetes, hypertension, asthma, and fatty liver disease^4^; and they also suffer from many social and psychological problems such as declined self-esteem and discrimination.^5,6^

Obesity was recently recognized as a disease by the American Medical Association.^7^ The etiology of obesity in children is complex and remains to be elucidated, with contributions from genetics, environment, lifestyle, and so on. While breastfeeding’s beneficial effect on many other outcomes, such as blood pressure and type 2 diabetes,^8^ has been well demonstrated, its relationship with childhood obesity remains unclear. A number of studies have been conducted to examine the influence of breastfeeding on childhood obesity. Earlier, systematic reviews and meta-analyses strongly supported the role of breastfeeding in reducing childhood obesity.^9–11^ However, later reviews and meta-analysis provided less supportive findings, labeling the effect of breastfeeding as small, tentative, or inconclusive.^8,12,13^ More recently, a study of 3271 children found that the influence of duration of breastfeeding on childhood body mass index (BMI) is trivially small, and statistically nonsignificant after adjusting for confounding factors.^14^ One large-scale trial of promoting breastfeeding also updated their results and found no association of breastfeeding with various measures of body composition.^15^ Given the inconsistencies surrounding the literature and the disagreement between the systematic reviews and meta-analyses, more research is needed to provide further evidence in examining the long-term effect of breastfeeding on childhood overweight and obesity, particularly those that control for confounding factors.

The China Family Panel Studies (CFPS) is an ongoing, prospective, and nationwide longitudinal study aiming to collect demographic, economic, educational, and health data to comprehensively explore the dynamic social changes in China.^16^ Using data collected by CFPS in 2010, in this paper we analyzed the association of breastfeeding with childhood bodyweight and obesity in China.

## METHODS

### Study Participants

CFPS is a comprehensive longitudinal social survey to collect individual, family, and community data to reveal the extensive changes that China has been undergoing. It was initiated in 2007, with 2 pilot studies conducted in 2008 and 2009, respectively. In 2010, it was formally implemented in 25 provinces/directly governed municipalities. A total of 14,960 families were interviewed, and the response rate exceeded 80%. Detailed information about the design and implementation of the studies has been reported elsewhere.^16^

CFPS thus includes information from a representative sample of individuals, families, and communities in China who will be followed in subsequent surveys, and therefore, provides valuable data for empirical analysis of a variety of social and economic studies. Parents’ help was sought in obtaining comprehensive information about the children. A total of 8990 children’s data were included in the study. This study was conducted according to the guidelines laid down in the Declaration of Helsinki and all procedures involving human subjects were approved by the Ethics Committee of Peking University. Written informed consent was obtained from all subjects.

### BMI and Assessment of Overweight and Obesity

Parents were asked to recall their children’s birth weight. BMI of the children at the survey was calculated using the self-reported height and weight information obtained from the survey. Overweight and obesity was defined, respectively, as ≥85th but <95th percentile and ≥95th percentile of BMI for children of the same age and gender.^17^ We define excessive body weight as being overweight or obese.

### Breastfeeding

Parents were asked to recall the number of months that the children were on breastfeeding. Unfortunately, no questions were asked regarding whether the child was breastfeeding exclusively and the duration of exclusive breastfeeding.

### Measurement of Other Confounding Factors

Children’s nationality and residence was provided by the parents and categorized into Han nationality versus others, and urban versus rural residence, respectively. Gestational age was provided by the parents through self-recall. Mother’s age at the child’s birth was calculated by subtracting the child’s age from the mother’s age in the survey. Mother’s education was self-reported and was categorized into less than high school, high school, and more than high school. Child’s health status was obtained using a proxy variable asking the number of doctor visits for the child in the last year, and was categorized into good (0 or 1 doctor visits), fair (2–4 visits), and poor (>4 visits). Mother’s emotional support to the child was estimated from the question that asked whether the parent actively participated in communication with the child and the responses categorized into 3 groups: strong (strongly agree or agree), fair (neural), and poor (disagree or strongly disagree). Mother’s health was obtained from self-report and categorized as good, fair, and poor. Mean income was calculated as the total income of the family divided by the number of family members.

Multiple imputation was employed to impute missing values for the included variables. Variables with a proportion of missing data >20% were excluded from further analysis, since it has been demonstrated that in such cases, multiple imputation cannot provide reliable results.^18^ As a result, mother’s self-reported health status was removed from subsequent analysis due to high proportion of missing data.

### Statistical Analysis

Chi-square or Fisher’s exact test was used when appropriate to compare the categorical variables by duration of breastfeeding. We performed multiple imputation for each variable with missing values. This was done using the Amelia II package,^19^ which imputes missing data based on the other variables in the dataset. This process was repeated 10 times, creating 10 imputed datasets.

We first treated duration of breastfeeding as a continuous variable (months). Following a previous study, we truncated breastfeeding at 12 months for children who received >12 months of breastfeeding.^14^ We then dichotomized the duration of breastfeeding in various ways and analyzed their influences on children’s obesity status: ever breastfeeding versus never breastfeeding, duration of breastfeeding ≥6 months versus <6 months, duration of breastfeeding ≥8 months versus <8 months, and duration of breastfeeding ≥12 months versus <12 months.

To analyze the effect of breastfeeding, we conducted unadjusted logistic regression, followed by adjusted logistic regression controlling for age, gender, nationality (Han vs others), residence (urban vs country), and weight at birth of the children, gestational age, mother’s age at delivery, parents’ evaluation of the children’s health, parental emotional support, and mean family income. This was done on the 10 imputed datasets using the Zelig package.^20,21^

Although we are aware of the existence of the generalized propensity score,^22^ which seems to be an appropriate statistical method for the analysis of our data, the severe skewness of duration of breastfeeding in our data violates the normality assumption for this method and prevents its application in our study, despite rigorous attempts to normalize the data. However, for each of the above dichotomization, we were able to perform propensity score matching^23^ in which matched cohorts were created after balancing the covariates to address differences in confounding factors. This can greatly reduce the risk of confounding. The 10 imputed datasets were matched using the nearest neighbor method in the MatchIt package.^24^ The matching quality was good (Figure S1, http://links.lww.com/MD/A29, jitter plot demonstrating the distribution of propensity score). The matched cohorts were then imported to the Zelig package and an adjusted logistic regression controlling for the aforementioned covariates was performed. For simplicity in the presentation, we mainly report the results using duration of breastfeeding ≥8 months versus <8 months. Results are similar for other dichotomization of duration of breastfeeding.

Previous studies reported that breastfeeding may have beneficial effect on the upper quantiles of the BMI distribution.^13^ To examine this potential effect, we performed quantile regression, a robust statistical method capable of revealing the association of a predictor with the response at a specific quantile,^25^ and therefore, is useful if the extreme values in the response are of particular interest. Propensity score was adjusted in the quantile regression. Quantile regression from the 10 imputed datasets yielded similar results.

Statistical analyses were performed using the program R (www.R-project.org) and Statistical Analysis System version 9.3 (SAS Institute Inc, Cary, NC).

## RESULTS

### Basic Characteristics of the Study Participants

This study included data from a total of 8990 children, among whom 1023 children had BMI ≤1 percentile or ≥99 percentile for children of the same age and sex and were excluded from analysis, leaving 7967 children with a mean age of 7.8 years (range 0–15). Table 1 presents the basic characteristics of the participants included in our analysis, stratified by duration of breastfeeding (≥8 month vs <8 months).

**TABLE 1:**
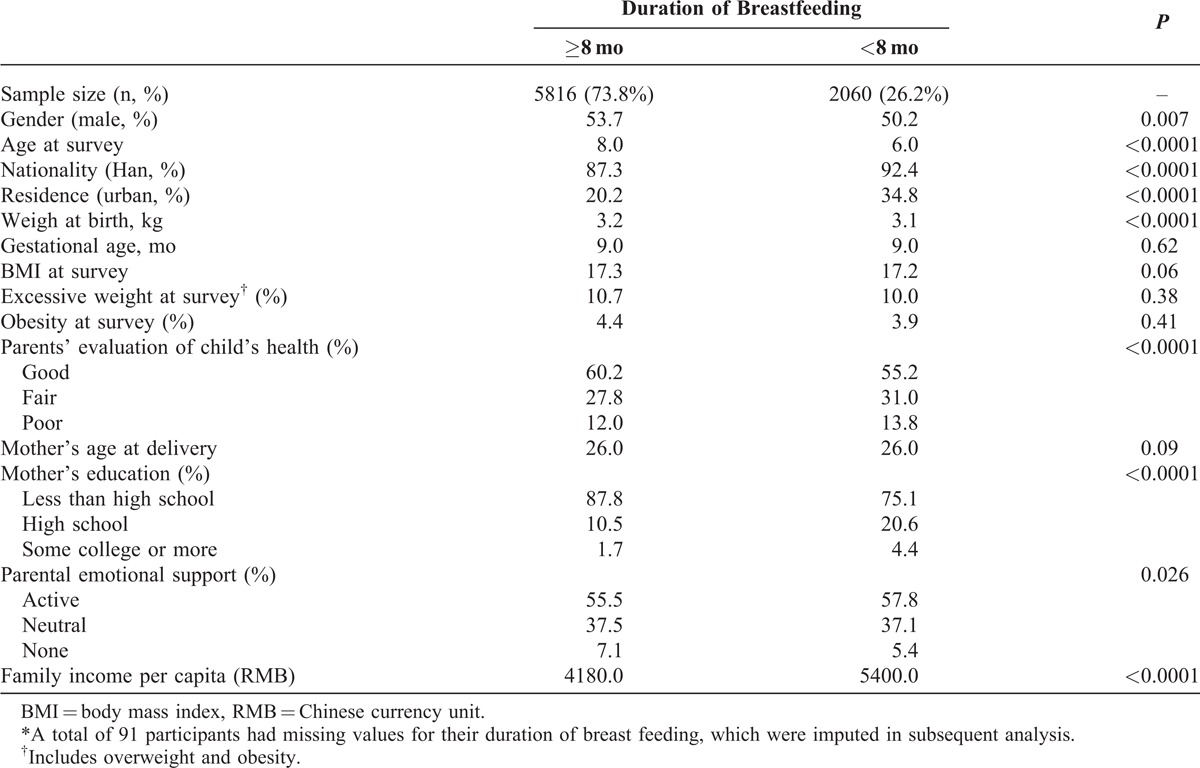
Basic Characteristics of the Study Participants by Duration of Breastfeeding (n = 7967)∗

The majority of children received >8 months of breastfeeding (73.8%). Children who are female, have Han nationality, live in urban regions, had a low birth weight, or live in a family with higher average family income were more likely to receive shorter duration of breastfeeding (Table 1). Mothers with higher education were more likely to offer shorter duration of breastfeeding (*P* < 0.0001), so were parents who had lower evaluation of their children’s health (*P* < 0.0001) and those who were more active in providing parental support (*P* = 0.026).

### Association of Breastfeeding With BMI

**FIGURE 1:**
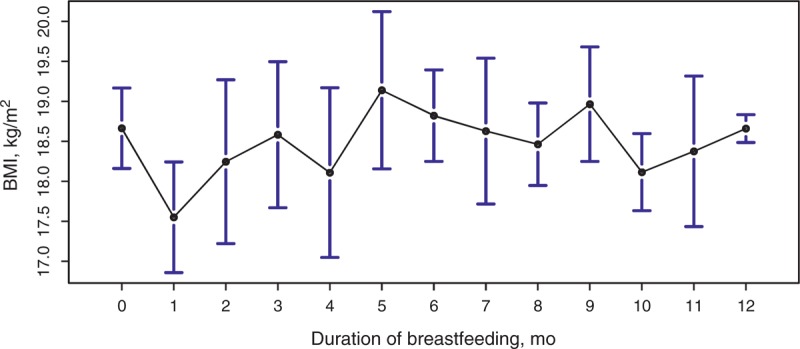
Mean BMI of children with different duration of breastfeeding. Duration of breastfeeding of 12 months includes all cases having duration of breastfeeding ≥12 months. The black circle indicates the mean, and the bars correspond to the 95% CIs.

There was no significant difference in BMI between those who had ≥8 months of breastfeeding and those who had <8 months of breastfeeding (17.3 vs 17.2, *P* = 0.06, Table 1). A detailed month-by-month examination revealed no tremendous variation in mean BMI with different durations of breastfeeding (Figure 1), except between those who received very short duration of breast feeding and those who received long duration of breastfeeding: children who receive ≥12 months of breastfeeding seem to have higher BMI than those who received only 1 month of breast feeding.

Quantile regression suggested that among children who have small to medium quantiles of BMI, longer duration of breastfeeding seems to be associated with higher BMI (Figure S2A and C–E, http://links.lww.com/MD/A30, difference in BMI for children with specific BMI quantiles; Table S1, http://links.lww.com/MD/A31, association of breastfeeding with BMI by quantile). There is a trend of decrease in BMI among children with larger quantiles of BMI (80%–90%), but the confidence interval (CI) is wide and there is no statistically significant result (Figure S2, http://links.lww.com/MD/A30, difference in BMI for children with specific BMI quantiles). Our analysis therefore fails to show beneficial effect of breastfeeding in children with extreme quantiles of BMI.

### Association of Breastfeeding With Childhood Obesity

**FIGURE 2:**
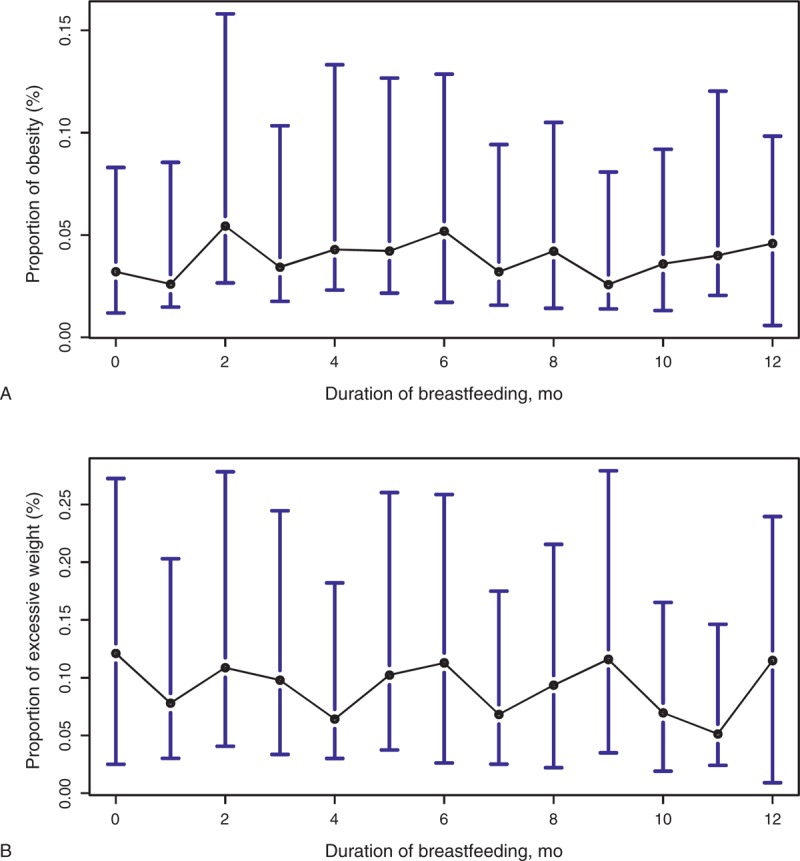
The proportion of obesity and excessive overweight among children with different duration of breastfeeding. Duration of breastfeeding of 12 months includes all cases having duration of breastfeeding ≥12 months. Excessive weight includes overweight and obesity. The black circle indicates the mean, and the bars correspond to the 95% CIs. (A) The proportion of obesity among children with different duration of breastfeeding. (B) The proportion of excessive weight among children with different durations of breastfeeding.

There is no statistically significant difference in the proportion of obesity with respect to different durations of breastfeeding (4.4% vs 3.9%, *P* = 0.41, Table 1). We did not find any significant association between duration of breastfeeding and childhood obesity from unadjusted or adjusted models (unadjusted odds ratio, OR = 1.02, 95% CI 0.99, 1.05, *P* = 0.12; adjusted OR 1.01, 95% CI 0.98, 1.05, *P* = 0.36; Table 2). Similar results were obtained after matching based on the propensity score. A detailed month-by-month examination revealed no dramatic change in the prevalence of childhood obesity (Figure 2A).

**TABLE 2:**
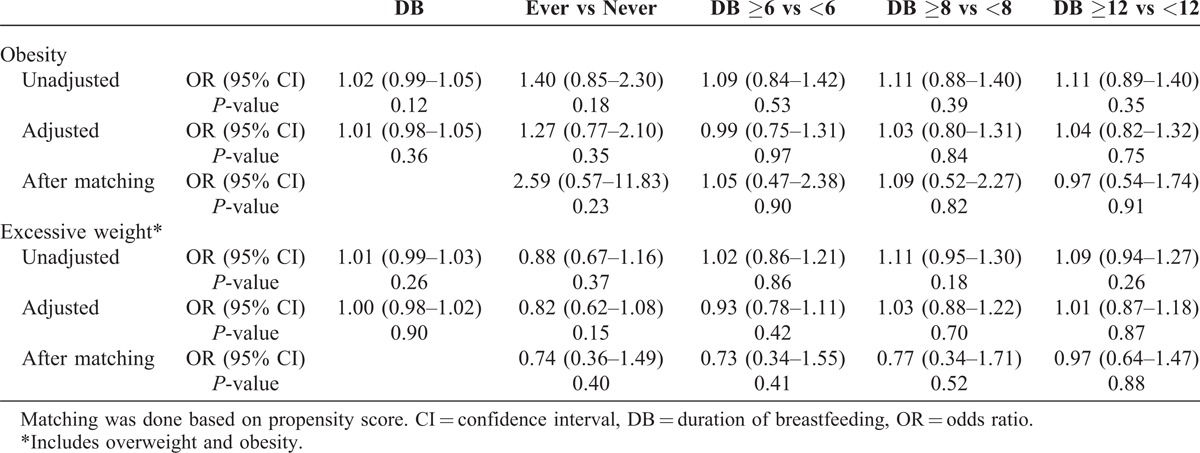
Association of Breastfeeding With Childhood Obesity

### Association of Breastfeeding With Childhood Excessive Weight

Children who had ≥8 months of breastfeeding are roughly equally likely to have excessive weight as those who had <8 months of breastfeeding (10.7% vs 10.0%, *P* = 0.38, Table 1). We did not find any significant association between duration of breastfeeding and excessive weight (unadjusted OR = 1.01, 95% CI 0.99, 1.03, *P* = 0.26; adjusted OR = 1.00, 95% CI 0.98, 1.02, *P* = 0.90, Table 2). Similar results were obtained after matching based on the propensity score. A detailed month-by-month examination revealed no dramatic change in the prevalence of childhood excessive weight (Figure 2B).

## DISCUSSION

In this paper, using data from the China Family Panel Studies conducted in 2010 in China, we analyzed the effect of breastfeeding on childhood BMI, obesity, and excessive weight. After adjusting for potential confounding factors or matching based on propensity score, we did not find any statistically significant effect of breastfeeding on reducing body weight, the risk of obesity, or excessive weight. We observed no beneficial effect among children with extreme BMI. Instead, among children who had small to medium quantile of BMI, we found that children tend to have higher BMI if they received longer duration of breastfeeding. To the best of our knowledge, this is the largest nationwide study in China investigating the potential effect of breastfeeding on obesity.

Previous studies have established the beneficial effect of breastfeeding to the human body, such as reducing the risk of infections^26^ and diabetes^27^ and promoting cognitive development.^28^ However, because the pathogenesis of overweight and obesity is complex involving factors including genetics, environment, and lifestyle, it is very challenging to disentangle the role that breastfeeding plays in contributing to overweight and obesity. Earlier, systematic reviews and meta-analyses found significant protective effect of breastfeeding on body composition.^9–11^ However, residual confounding cannot be ruled out in these studies. And they are also subject to publication bias and heterogeneity among the studies. These researches often included studies with different definition of overweight and obesity and with different adjustment for confounding factors, and they focused on the assessment of the relationship with overweight and obesity. Another separate meta-analysis by the same authors of one of the above meta-analyses investigated the effect of breastfeeding on mean BMI, but did not find significant effect after adjustment for certain covariates.^29^ This motivated the examination of the effect of breastfeeding among children with upper percentiles of the BMI distribution. A recent study examined 14,412 children and identified a potential protective effect of breastfeeding: children with BMI values between 90th and 97th percentiles had a significance reduction in mean BMI if they had breastfeeding compared with bottle feeding.^13^ We did not find a significant change in mean BMI in children who had medium to high quantiles of BMI, and children who had small to medium quantiles of BMI seem to have higher BMI if they had longer duration of breastfeeding (Figure S2, http://links.lww.com/MD/A30, difference in BMI for children with specific BMI quantiles). The mechanism underlying this interesting phenomenon and the reasons accounting for the inconsistencies of the findings deserve further investigation.

More recent studies provided less consistent conclusions regarding the association of breastfeeding with body composition. The meta-analysis by the World Health Organization found that breastfeeding significantly reduced the risk of overweight/obesity (OR = 0.78, 95% CI 0.72–0.84).^8^ However, many issues concerning the methodology employed in this meta-analysis have been identified, challenging the conclusion of the study.^30^ A recent study analyzed the duration of breastfeeding and child obesity via a generalized propensity score approach, taking into account many confounding factors. Significant result was found only in the unadjusted model, and the significance disappeared once covariates were included.^14^ Another study found significant effect of breastfeeding in reducing BMI in children aged <5 years, but not in children aged 5–6 years.^31^ Our study adds further evidence to support the conclusion of these latter studies showing no significant beneficial effect of breastfeeding in reducing BMI.

Interestingly, in our study, there seems to be a trend toward higher risk of being obese with longer duration of breastfeeding (Figure 2A). There seems to be an increasing trend in the prevalence of obesity if breastfeeding was provided for 9 months or longer. A similar but nonsignificant trend for BMI was also found in one of the multiple studies included in a recent research,^32^ and in a meta-analysis comprising 5 prospective cohorts in low-middle income countries.^33^ Similar trend for overweight and obesity was found in an earlier study that reported that children who had breastfeeding for >11 months seemed to be more likely to be overweight and obese compared to those who had 6–11 months of breastfeeding.^34^ However, these results should be interpreted cautiously as they do not reach statistical significance, and do not differentiate between exclusive breastfeeding and breastfeeding with complementary food. Duration of predominant breastfeeding was associated with a decreased likelihood of being obese at 18 years old.^34^ Moreover, these results might be confounded by practices of complementary feeding (CF). For example, late introduction of CF, rather than the duration of breastfeeding, was shown to be protective of adult overweight.^35^ And during infancy and early childhood, there is a high frequency of inappropriate/inadequate CF,^36^ which may be associated with various diseases/disorders in later life, including obesity.^37^

Most researches on the association of breastfeeding with body composition are observational studies because it is infeasible and unethical to conduct interventional studies that randomly assign breastfeeding to mother–infant pairs. However, it is ethical to randomly assign breastfeeding promotion. The promotion of breastfeeding interventional trial represents one of the few such trials.^38^ Although the study has identified protective effect of breastfeeding in various aspects,^38,39^ it did not find any significant difference in mean BMI, prevalence of overweight, and obesity between the intervention and the control group.^40^ The group recently updated their research but found similar null association of breastfeeding with a variety of measurements for body composition among participants with a median age of 11.5 years.^15^ Lack of power is an important issue for such interventional studies. Previous researches indicate that the effect of breastfeeding on body composition, if it exits, might be very small. It was calculated that for intervention studies with 8000 in each arm, the power for detecting a difference of 0.04 in BMI is only 4%, implying that it is extremely unlikely for a single study to recruit enough participants to gain sufficient power (eg, ≥75% power).^13^ More individual studies that control for confounding are warranted such that future meta-analysis can utilize these studies to achieve satisfactory statistical power.

China has a special culture that is very different from that of many other western counties and might play an important role in accounting for the increasing prevalence of child overweight and obesity in China. There have been studies conducted in China investigating the contribution of breastfeeding to overweight and obesity. An early study conducted in 8 cities in China found that breastfeeding was not associated with obesity among children aged <7 years old.^41^ A study in Beijing reported children who were breastfed for at least 4 months had significantly reduced risk of being overweight.^42^ However, the possibility of residual confounding cannot be ruled out as the study was restricted to Beijing, a relatively modernized city in China. Another community-based study found significant protective effect of breastfeeding in reducing weight gain and BMI in the first 3 months of urban Chinese infants.^43^ However, another study conducted in Hong Kong failed to demonstrate the association of breastfeeding with mean BMI among the “children of 1997” birth cohort.^44^ More studies in China are needed to further establish the relationship of breastfeeding with body composition in China.

There are limitations with this study. Duration of breastfeeding was obtained through recall and, therefore, is subject to recall bias. As the CFPS is not targeting specifically breastfeeding, we did not have information about the formula used during breastfeeding and thus could not differentiate between exclusive breastfeeding and breastfeeding combined with formula feeding. BMI was calculated through mother-reported height and weight, and therefore may not be as accurate as anthropometric measurements. However, we believe that the bias is not systematic given the random nature of the sampling design of CFPS and the large sample size of this study. There are other measures for assessment of body composition, such as whist–hip ratio for measuring abdomen obesity, but these types of data were not collected in CFPS, and we could not examine the effect of breastfeeding on them. This study is also limited due to its cross-sectional nature, and we expect that with the availability of longitudinal data collected in future CFPS surveys that track infant participants as they grow, we can have more conclusive results. Finally, there are other confounding factors that have been found to be related to BMI by prior literature but were not collected by CFPS, such as maternal smoking.^45^ However, given the negative results of this study, our finding is unlikely confounded by these factors. It is possible that breastfeeding may have significant effect on bodyweight in later rather than early life. Limitations of our data prevent us from investing this possibility.

In summary, although breastfeeding has many advantages to the human body, our research finds no significant protective effect in reducing BMI or the risk of obesity or excessive weight in children in China. This adds further evidence against any population-wide strategy in attempting to reduce obesity through promotion of breastfeeding.
